# Implementing stereotactic arrhythmia radioablation with STOPSTORM.eu consortium support: intermediate results of a prospective Israeli single-institutional trial

**DOI:** 10.1007/s00066-024-02300-z

**Published:** 2024-09-16

**Authors:** Egor Borzov, Roi Efraim, Mahmoud Suleiman, Raquel Bar-Deroma, Salem Billan, Jingyang Xie, Stephan Hohmann, Oliver Blanck, Tomer Charas

**Affiliations:** 1 Department of Radiotherapy, Rambam Health Care Campus, Haifa, Israel; 2 University of Lübeck, Institute for Robotics and Cognitive Systems, Lübeck, Germany; 3 Department of Cardiology and Angiology, Hannover Heart Rhythm Center, Hannover Medical School, Hannover, Germany; 4 Department of Radiation Oncology, University Medical Center Schleswig-Holstein, Kiel, Germany

**Keywords:** Ventricular tachycardia, Cardiac radioablation, STAR, Refractory VT, RAVENTA clinical trial

## Abstract

**Background:**

Ventricular tachycardia (VT) is a life-threatening arrhythmia originating from the heart’s ventricles. Traditional treatments include antiarrhythmic medications, implantable cardioverter-defibrillators (ICDs), and catheter ablation. Stereotactic body radiation therapy (SBRT) targeting the arrhythmogenic focus in the left ventricle—stereotactic arrhythmia radioablation (STAR)—is an emerging treatment and may offer a potential solution for patients with refractory VT.

**Objective:**

We designed an interventional prospective clinical trial in Israel aligned with the STOPSTORM.eu consortium’s benchmarks, recommendations, and directives to assess the safety and efficacy of STAR in patients with refractory VT.

**Methods:**

Our phase I/II single-institutional trial was approved by the Ministry of Health of Israel for 10 patients, initially assessing safety in the first 3 patients. We included patients with ICDs experiencing symptomatic monomorphic VT after an inadequate response to previous therapies. The primary endpoints were treatment-related serious adverse events and a reduction in VT burden as assessed by ICD interrogation. Secondary outcomes included a reduction in antiarrhythmic medications and changes in quality of life.

**Results:**

From August 2023 to August 2024, 3 patients underwent STAR treatment. The prescription dose was a single fraction of 25 Gy. Planning target volumes were 47.8, 49.7, and 91.8 cc, and treatment was successfully delivered with no grade 3 or higher adverse events reported. Over a follow-up period of 12 months for the first patient and 8 months for the second one, no VT events were recorded after treatment. The third patient died from progressive heart failure 3 months after treatment. Left ventricular ejection fraction remained stable, and no significant radiation-induced inflammatory changes were noted.

**Conclusion:**

The initial results of this trial suggest that STAR can reduce VT episodes in patients with refractory VT without severe adverse effects. The study highlights the importance of international collaboration and standardization in pioneering new treatments. Further follow-up and additional patient data will be necessary to confirm these findings and evaluate long-term outcomes, including potential adjustments to antiarrhythmic medication regimens.

## Introduction

Ventricular tachycardia (VT) is a cardiac arrhythmia characterized by a rapid and abnormal heartbeat originating from the heart’s ventricles, potentially leading to sudden cardiac death. VT can be managed through various approaches depending on the severity, frequency, and underlying causes of the arrhythmia. Treatment options may include antiarrhythmic medications, implantable cardioverter-defibrillator (ICD) placement to deliver antitachycardia pacing (ATP) or electrical shock to restore normal rhythm, and catheter ablation [1]. Catheter ablation is a procedure that applies energy through radiofrequency waves, pulses, or cryoablation to targeted areas of the heart. Its purpose is to destroy abnormal electrical pathways and decrease or prevent episodes of ventricular tachycardia. However, despite the generally high effectiveness of these treatments, there are instances in which catheter ablation is technically infeasible or fails to sufficiently suppress the VT and patients may experience recurrences of the arrhythmia [2].

Stereotactic arrhythmia radioablation (STAR) is a newer approach to treating ventricular tachycardia that utilizes a stereotactic body radiation therapy (SBRT) approach to target the origin of VT in the left ventricle [3]. It is supposed that a higher radiation dose (> 30 Gy) induces cellular damage which may lead to elimination or functional impairment of the arrhythmogenic source [4]. Another effect may be achieved by moderate radiation doses (15–25 Gy), where high-energy radiation delivered to the arrhythmogenic substrate causes disruption of cellular electrophysiology, potentially altering gene expression levels, changing ion channel function, and reducing the activity for arrhythmia generation by increasing conduction velocity [5]. However, the biological and cellular mechanisms of STAR are still widely discussed [6]. Nevertheless, STAR can be considered as a bail-out option for patients with recurrent or refractory VT who have failed conventional therapies [7].

Since a pioneering in-patient study in 2017 [8], STAR has received enormous interest in the medical community and has accelerated multiple national clinical trials around the world [9]. However, due to the high complexity of STAR, including integration of advanced imaging and electrophysiological mapping into radiation treatment planning, complex motion management with precise treatment delivery, and patient safety, published results have been practically difficult to unify and analyze to obtain reliable conclusions until now. In the European Union, this led to foundation of the Standardized Treatment and Outcome Platform for Stereotactic Therapy of Re-Entrant Tachycardia by a multidisciplinary consortium (STOPSTORM.eu) to create a unified database to optimize and harmonize STAR treatments [10].

The aim of our national study in Israel was to design an interventional prospective clinical trial in full accordance and coordination with the STOPSTORM.eu consortium and its benchmarks, recommendations, and directives regarding each step of STAR treatments. The purpose of this paper is to share our study design and report intermediate clinical outcomes per protocol for the first patients.

## Materials and methods

### Study design and endpoints

This phase I/II single-institutional trial was designed based on the assessment of the vulnerability of patients suffering from refractory VT with limited treatment options [11] and modelled after the STOPSTORM national trial in Germany (RAVENTA [4]). The key inclusion criteria for the trial are patients with an implanted ICD who suffered from symptomatic monomorphic ventricular tachycardia with low effectiveness or failure of previous local ablative therapy [12]. The key exclusion criteria are a lack of evidence of myocardial scar triggering VT or other radiosurgery contraindications such as pregnancy or prior thoracic irradiation. The post-STAR follow-up of the patient is conducted by a radiation oncologist and a cardiologist for 3 years to register further short- and long-term side effects and the duration of response. Data on VT burden and ICD therapies are obtained via remote monitoring and ICD interrogation during each study visit.

The trial was approved by the Ministry of Health of Israel and the institutional review board (national trial number: 202225964) for the first 3 patients for an interim safety analysis and a total of 10 patients after permission to continue enrollment. The Ministry of Health statistical requirement for the primary safety outcome was that there must be no grade 3 treatment-related complications for more than one out of every 3 patients, which is in general accordance with RAVENTA [4]. The primary efficacy outcome was a reduction in VT burden as assessed by ICD interrogation. Reduction of antiarrhythmic medications and change in quality of life as measured by questionnaire were set as secondary efficacy outcomes.

### Treatment workflow

#### Baseline and cardiac imaging

Initial evaluation of the patient’s eligibility was performed by a cardiologist based on inclusion/exclusion criteria, transthoracic echocardiography, left ventricular ejection fraction, and the patient’s performance status. After obtaining the patient’s consent to participation, intravenous contrast-enhanced cardiac CT (CCT) was performed to define heart sub-structures, myocardial scarring, and wall-thinning areas as per STOPSTORM recommendations [13]. Per the study protocol, additional imaging modalities such as cardiac MRI, PET/CT, or SPECT/CT were optional to better characterize the arrhythmogenic substrate.

#### Target definition by electroanatomical mapping

The target region was primarily defined from the electroanatomical mapping (EAM) procedure and included the area defined by the treating electrophysiologist as the VT substrate that would undergo catheter ablation [14]. All EAM procedures were performed under general anesthesia. For navigation during EAM, 3D registration of the EAM geometry with the CCT model was accomplished via identifiable anatomic reference points (coronary sinus, aortic arch, and LV apex) as landmarks for initial alignment and automatic surface registration using CartoMerge (Biosense Webster, Irvine, CA, USA).

Mapping was performed for each patient in order to define the target volume. A 6-French decapolar catheter was inserted from the right femoral vein and placed into the right ventricle. The left ventricle (LV) endocardium was accessed by a transseptal or retrograde transaortic approach. EAM during sinus rhythm was performed using the CARTO (Biosense Webster) mapping system. Mapping was performed with a 3.5-mm irrigated-tip catheter (THERMOCOOL SMARTTOUCH™ SF, Biosense Webster) and a multipolar high-density mapping catheter (OCTARAY™, Biosense Webster). Bipolar signals were filtered from 30 to 250 Hz. The following voltage criteria were used: a peak-to-peak bipolar amplitude of < 1.5 mV, defined as the total low-voltage zone; an amplitude of 0.5 to 1.5 mV, the border zone; and an amplitude of < 0.5 mV dense scar. Local abnormal ventricular activity (LAVA) was identified by the presence of low-voltage, high-frequency electrograms distinct from the far field ventricular electrogram, sometimes displaying double or multiple components separated by very-low-amplitude signals or an isoelectric interval. Pace mapping was performed from these sites to identify the potential exit and ablation target. Since target definition can vary significantly for STAR [15], a group consensus of the local study team together with experts from the STOPSTORM.eu consortium was achieved for the VT substrate definition.

#### Target transfer to radiation oncology

The outlined/tagged VT substrate of the endocardial surface area was then transferred to the CCT under quality assurance (QA) guidance of a dedicated registration and visualization software for STAR developed within the STOPSTORM/RAVENTA framework (CARDIO-RT, University of Lübeck, Germany [16]). For QA, the 2D/3D registration method between the EAM screenshots and the endocardial LV was used as previously described [16]. The target as outlined by the EAM was mapped onto the endocardial LV surface and transmurally expanded into the LV myocardium. The resulting volume is called the target volume (TV) and serves a purpose similar to that of a clinical tumor volume (CTV) in standard radiotherapy treatments, meaning that it is the main area to be treated by radiation.

#### Motion management

Since TV motion in the left ventricle can vary significantly per patient [17], the motion management for STAR was implemented based on expert advice from the STOPSTORM.eu consortium and based on the patient’s clinical situation as previously described [18]. The primary imaging for radiation treatment planning (simulation CT, SCT) was carried out in the treatment position as implemented for regular lung stereotactic body radiotherapy (SBRT) [19]. A time-resolved, breathing-motion-gated 4D SCT was performed to account for respiratory motion, while the previously acquired CCT was for heart beating motions, accordingly. For patient immobilization during a treatment, the vacuum BlueBAG BodyFIX (Elekta AB, Stockholm, Sweden) was used and, if clinically necessary, an abdominal compression device can be used [20]. Subsequent registration of CCT and averaged 4D SCT was performed, allowing the TV structure to be transferred to the SCT. According to 4D SCT and the CCT motion data, the TV was expanded to the internal target volume (ITV), and the latter was expanded to the planning target volume (PTV), applying technical treatment margins based on imaging data and departmental and international lung SBRT recommendations and guidelines [21].

#### Beam technique planning and treatment delivery

Prescribing, recording, and reporting of STAR treatment were in full accordance with ICRU91 recommendations [22]. STAR was performed as a single-fraction radiotherapy treatment with a prescription dose of 25 Gy. Organs at risk (OARs) limitations were based on RAVENTA and STOPSTORM recommendations [4]. In specific cases, if treatment efficacy is clinically prioritized for STAR and heart substructures such as coronary arteries and valves are located inside the PTV, the major dose deviations up to the near maximum dose of 25 Gy were accepted. Treatment planning was performed using Monaco TPS (Elekta AB, Sweden) and the plan was delivered with a Versa HD (Elekta AB, Sweden) linear accelerator using a 6-MV flattening-filter-free beam in VMAT mode. Image guidance was performed with the help of 4D cone-beam CT, and positioning was aided by the Hexapod 6D system (Elekta AB, Stockholm, Sweden). STAR was delivered under the direct on-site supervision of the cardiologist and the radiation oncology staff while the ICD was turned off and the patient was connected to the monitoring system.

#### Blanking period and follow-up

The post-STAR follow-up of the patient is conducted by a radiation oncologist and a cardiologist for 3 years to register further short- and long-term side effects and the duration of response. Safety was assessed by means of echocardiography and chest X‑ray at day 1 and at 2 weeks after treatment. A 6-week post-treatment period was set as a blanking period where arrhythmias may occur because of inflammation or delayed effects of the irradiation. Data on VT burden and ICD therapies were monitored by periodic ICD interrogation as part of clinical care to assess the treatment efficacy.

## Results

From August 2023 to July 2024, 3 patients underwent STAR according to our protocol. Table 1 illustrates the demographic and clinical data for each patient. Two of the selected patients were diagnosed with nonischemic cardiomyopathy and one with combined valvular and ischemic cardiomyopathy. All patients had heart failure with reduced ejection fraction: 15%, 30%, and 27.5%, respectively. Patients 1 and 2 were taking antiarrhythmic drugs (AAD) at the time of evaluation, while patient 3 had amiodarone-related thyrotoxicosis and the drug was withdrawn a few years ago. For all patients, the clinical VT persisted despite AAD treatment. Additionally, before STAR treatment, all patients underwent invasive catheter ablation procedures without satisfactory long-term results and eventual recurrence of ventricular arrhythmia. For each patient, CCT and EAM (without ablation) procedures were performed closely prior to STAR treatment to update data and precisely define the VT source.

**Table 1:** Demographic and clinical characteristics of the patients. NYHA New York Heart Association

Parameter	Patient 1	Patient 2	Patient 3
Age	64	63	72
Sex	Male	Male	Male
Type of cardiomyopathy	Nonischemic	Nonischemic	Ischemic
NYHA class	IV	II	IIIA
Antiarrhythmic medication	Amiodarone, sotalol, mexiletine	Amiodarone	Amiodarone
No. of previous catheter ablations	1	4	2
No. of VT episodes 12 months before STAR	5	15	6
STAR planning target volume, cc	48	50	92
Treatment session duration {beam-on time}, minutes	30 {11}	30 {10}	30 {8}
Hospitalization after STAR, days	7	5	10
Follow-up time, months	12	8	3^a^
No. of VT episodes during Follow-up {during the 6‑week blanking period}	0	0	12 {1}
*Left ventricular ejection fraction, %*			
Prior STAR	15	30	27.5
2 weeks after	17.5	35	30
6 weeks after	17.5	35	–^a^

^a^Patient died 3 months after the treatment

**Fig. 1:**
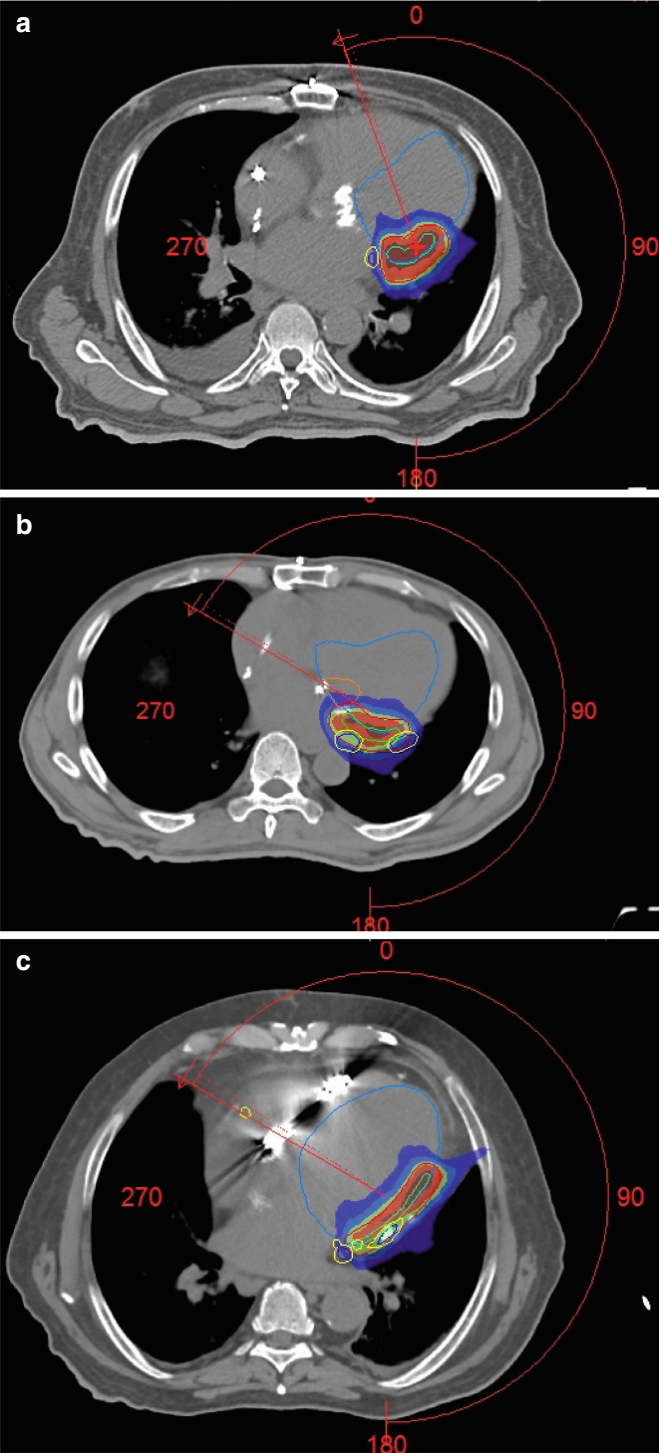
Examples of the STAR treatment plans. Dose sparing of coronary sinus (CS) and coronary arteries (CA; **a**, **b**) and the mitral valve (**c**). Structures: *blue* left ventricle, *dark blue* CS and CA, *yellow* 2-mm margin of CS and CA, *orange* mitral valve, *red* Planning Target Volume PTV, *green* Target Volume TV. Doses: *dark blue* 15 Gy, *blue* 19 Gy, *green* 22.5 Gy, *red* 25 Gy, *dark red* 28 Gy

The treatment and planning parameters are summarized in Table 2. Target volumes were 10.3, 7.4, and 17.4 cc and corresponding planning target volumes 47.8, 49.7, and 91.8 cc. The treatment was successfully delivered in all patients and beam-on times were 11, 10, and 8 min. An example of the treatment plan dose distribution is shown in Fig. 1. A partial VMAT arc of 240 degrees was used. This case demonstrates the trade-off between PTV coverage and dose constraints for the coronary sinus (CS) and coronary arteries (CAs). Applying a 2-mm safety margin to the CS and CAs, the dose was limited in this region to up to 25 Gy—the compromise dose was expected to be sufficient for the effect of the STAR procedure to be achieved without damaging the coronary arteries. At the same time, in the internal core regions of the TV, in the absence of critical structures, the dose was escalated to up to 30 Gy.

**Table 2:** Treatment plan characteristics. TV Target Volume. PTV Planning Target Volume

Parameter	Patient 1	Patient 2	Patient 3
Treatment time, min	11	10	8
TV, cc	10.3	7.7	17.4
PTV, cc	47.8	49.7	91.8
PTV D98%, Gy	24.6	19.5	23.2
PTV D50%, Gy	27.8	25.4	26.9
PTV D2%, Gy	31.5	28.5	29.0

By June 2024, no grade 3 or higher radiation-related adverse events had been registered. During the follow-up period, patient 1 underwent successful trans-catheter edge-to-edge mitral valve repair (TEER) due to deterioration of mitral valve regurgitation, with significant clinical improvement. Patient 3, who has combined valvular and ischemic cardiomyopathy, underwent transcatheter aortic valve replacement back in 2014. In 2019, the mass on the prosthetic valve was diagnosed as a thrombus; however, after further evaluation following the STAR procedure, the mass is suspected to be related to Q fever endocarditis. After a number of consequent recurrent heart failure hospitalizations, patient 3 died from progressive heart failure.

**Fig. 2:**
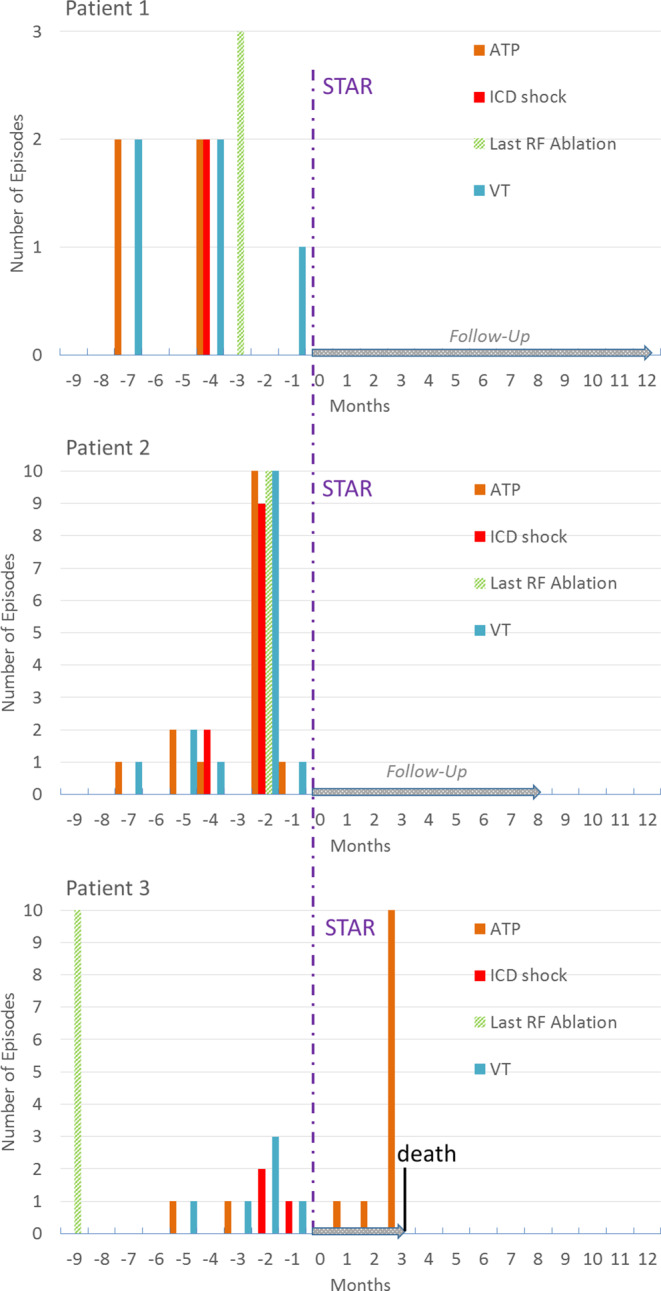
Timeline of the events: 0 is the moment of the STAR procedure; positive is months during follow-up. Patient 3 died 3 months after STAR. ATP Antitachycardia Pacing, ICD Implantable Cardioverter-Defibrillator, RF Radio Frequency, VT ventricular Tachycardia

Figure 2 shows a timeline of VT events, ICD shocks, antitachycardia pacing (ATP), catheter ablations, and STAR treatment on a per-patient basis. Patients 1 and 2 have had no VT events until now (12 and 8 months, correspondingly). Patient 3 had 12 consecutive VT events 3 months after therapy: one during the blanking period, one 2 months after STAR, and 10 consequent ones during 48 h—10 days prior to his death. All events were terminated after an average of 8 s with a single ATP delivery.

Additionally, no ICD system performance errors were noted. A series of echocardiography and chest X‑ray examinations did not demonstrate any radiation-induced inflammatory changes. The mean change in left ventricular ejection fraction is shown in Table 1.

For the first and second patients, the STAR procedure was performed under current AAD treatment and even though the patients can be assessed as clinically free from VT events during follow-up, the cardiologist decided not to stop/change the antiarrhythmic drug until the last time of evaluation. Since the patients were very refractory to AAD and RF ablation, cardiologists decided to follow them carefully for at least 1 year before changing the AAD, unless side effects enforce it.

## Discussion

In this paper, we describe the study design for a single-institution STAR phase I/II trial and report the intermediate clinical results. The patients were selected for this treatment because they had ventricular tachycardia (VT) nonresponsive to antiarrhythmic drugs and refractory to previous invasive catheter ablations. Such patients with a recurrence of VT have a poor prognosis, suffer from recurrent ICD shocks, and have limited or no further treatment options to reduce the burden of VT. To provide a bail-out treatment option for such patients, a dedicated STAR trial has been launched in our country.

Regarding this new procedure, it is well known that due to the complexity and variability of STAR and the lack of extra-institutional standardization, the majority of previous case series and even data reported from clinical trials are hardly compatible [10]. As a solution, the STOPSTORM.eu consortium has been established to investigate STAR and provide a treatment database to evaluate patterns of practice and outcomes of this novel procedure and ultimately to harmonize STAR within Europe [10]. For non-EU medical centers, due to the limited clinical experience and rapidly increasing learning curve, it is important to run such a trial in cooperation with experienced medical centers. The STOPSTORM consortium kindly offered its support, which brings significant benefits for both sides, particularly for the patients. Our trial has been designed in full accordance with STOPSTORM benchmarks and recommendations and aligned with one of its national trials (RAVENTA, Germany) [4]. Furthermore, the Israeli National Ministry of Health approved the current trial for the first 3 patients as an initial step, and after reporting positive results, the trial will continue recruiting patients. This procedure was like that of the RAVENTA trial, where intermediate results had to be reported after 5 patients [23]. Because of a small patient cohort of three cases, which limits making statistically significant conclusions, we report the intermediate results using descriptive statistics.

During follow-up, no grade 3 or worse STAR-related severe side effects were noticed. This is in general agreement with data from recent studies reporting that STAR can be safely applied to selected patients with a low profile of grade 3 toxicity during a 30-day post-radiosurgery timeframe [23]. LVEF did not worsen as a measure of safety and no radiation-induced inflammation was noted after treatment. On the contrary, new research has studied the possible positive effect of increasing LV ejection fraction and ventricular function after whole-cardiac low-dose irradiation of about 5 Gy [24]. The mean doses for the heart minus the PTV were 3.4, 4.6, and 7.4 Gy in our patients and further changes in LVEF will be monitored. As noted earlier, patient 3 passed away 3 months after the STAR procedure. It is supposed that the deterioration leading to his death was due to an uncontrolled Q fever infection, compounded by severe heart failure and other comorbidities, and that the recurrent arrhythmia episodes observed in the final weeks before his death were indicators of worsening heart failure.

The metrics for measuring the efficacy of STAR treatment are still not well established. The evaluation of efficacy may encompass a wide spectrum of procedural goals: from emergency bail-out procedures for patients in electric storm to reducing the number of ICD shocks or simply diminishing premature ventricular contractions (PVCs) [25, 26]. Therefore, the combination of each patient’s individual specific conditions and the procedural goals define the relevant parameters for assessing the efficacy of STAR. In our trial, the reduction of VT burden is primarily evaluated through a decrease in VT events and ICD interventions, which include antitachycardia pacing (ATP) and shocks. As seen in Fig. 2, patient 1 and patient 2 experienced a 100% reduction in VT events, while patient 3 had 12 events of ATP-managed VT during follow-up. Based on the most current systematic review and meta-analysis of prospective trials, the expected outcome should be a VT reduction of more than 95% for 60–80% of patients [27]. At the same time, parameters such as the 1‑year recurrence-free rate and 1‑year recurrence-free survival rate have huge deviations between different studies, which might be due to population inhomogeneity or even treatment methodology [28, 29].

Before STAR, during follow-up with a cardiologist, all 3 patients had severe side effects, and cardiologists intended to withdraw or decrease the AAD, especially amiodarone. After STAR, the electrophysiologists decided to not decrease or withdraw AAD due to the relatively short duration of follow-up and the fact that in the past, selected patients had had many episodes of VT with multiple failed treatments. The decision of whether to decrease or withdraw antiarrhythmic medications will be taken further on an individual basis.

Another important clinical outcome is the patient’s quality of life (QoL), which is reduced with ongoing VT. Patients were planned to report QoL questionnaires three times: baseline before treatment and at 3 and 12 months after treatment. It is expected that a rather long ICD shock-free period must pass for a patient’s anxiety level to decrease. Because of limited follow-up, we did not present the results of the SF-36 analysis and decided to wait for the 1-year questionnaire.

The main limitation of this report on an ongoing clinical trial is the limited follow-up. Intermediate results do not allow performance of statistical analysis. Patients were recruited for 1 year, which is longer than initially expected. After the intermediate safety assessment by the Ministry of Health, the enrollment will continue for the next 7 patients.

## Conclusion

In this paper, we have reported the intermediate results of our STAR trial which has been set up with the support of the STOPSTORM.eu consortium. The current data show that in our patients with refractory VT and limited treatment options, stereotactic arrhythmia radioablation (STAR) as a bail-out therapy resulted in a remarkable reduction in VT episodes, while no treatment-related side effects were observed. The secondary efficacy outcome of antiarrhythmic medication reduction has not yet been reached due to the cardiologist’s decision at this time and will be further investigated.
